# Estimating heart mass from heart volume as measured from post-mortem computed tomography

**DOI:** 10.1007/s12024-022-00478-1

**Published:** 2022-04-28

**Authors:** Hamish M. Aitken-Buck, Matthew Moore, Gillian A. Whalley, Larissa Lohner, Benjamin Ondruschka, Sean Coffey, Rexson D. Tse, Regis R. Lamberts

**Affiliations:** 1 Department of Physiology, HeartOtago, School of Biomedical Sciences, University of Otago, Dunedin, 9054 New Zealand; 2 Department of Medicine, HeartOtago, Dunedin School of Medicine, University of Otago, Dunedin, New Zealand; 3 Institute of Legal Medicine, University Medical Center Hamburg-Eppendorf, Hamburg, Germany; 4 Department of Forensic Pathology, LabPLUS, Auckland City Hospital, Auckland, New Zealand

**Keywords:** Heart mass, Heart volume, Computed tomography, Post-mortem

## Abstract

Heart mass can be predicted from heart volume as measured from post-mortem computed tomography (PMCT), but with limited accuracy. Although related to heart mass, age, sex, and body dimensions have not been included in previous studies using heart volume to estimate heart mass. This study aimed to determine whether heart mass estimation can be improved when age, sex, and body dimensions are used as well as heart volume. Eighty-seven (24 female) adult post-mortem cases were investigated. Univariable predictors of heart mass were determined by Spearman correlation and simple linear regression. Stepwise linear regression was used to generate heart mass prediction equations. Heart mass estimate performance was tested using median mass comparison, linear regression, and Bland–Altman plots. Median heart mass (*P* = 0.0008) and heart volume (*P* = 0.008) were significantly greater in male relative to female cases. Alongside female sex and body surface area (BSA), heart mass was univariably associated with heart volume in all cases (*R*^2^ = 0.72) and in male (*R*^2^ = 0.70) and female cases (*R*^2^ = 0.64) when segregated. In multivariable regression, heart mass was independently associated with age and BSA (*R*^2^ adjusted = 0.46–0.54). Addition of heart volume improved multivariable heart mass prediction in the total cohort (*R*^2^ adjusted = 0.78), and in male (*R*^2^ adjusted = 0.74) and female (*R*^2^ adjusted = 0.74) cases. Heart mass estimated from multivariable models incorporating heart volume, age, sex, and BSA was more predictive of actual heart mass (*R*^2^ = 0.75–0.79) than models incorporating either age, sex, and BSA only (*R*^2^ = 0.48–0.57) or heart volume only (*R*^2^ = 0.64–0.73). Heart mass can be more accurately predicted from heart volume measured from PMCT when combined with the classical predictors, age, sex, and BSA.

## Introduction

Changes in heart mass are well established as an indicator of cardiovascular pathology and cardiovascular-related death at post-mortem examination [1]. For instance, an increased heart mass provides a non-specific indication cause of death associated with cardiac conditions, such as cardiomyopathy, valvular disease, or sudden cardiac death [1]. Conventionally, heart mass is measured invasively from the absolute weight of the dissected heart and compared to established reference ranges. In recent decades, however, estimating heart mass from non-invasive imaging, in particular post-mortem computed tomography (PMCT) or cardiac magnetic resonance imaging (cMRI), has become more common in post-mortem examination [2]. This approach is appealing since the data can be stored permanently and re-examined without compromising tissue integrity [2]. Moreover, predicting cause of death less invasively provides an alternative examination strategy to interested parties that might be reluctant to pursue invasive autopsy [2].

Apart from cardiovascular pathology, heart mass is independently associated with the age and sex of post-mortem cases, as well as with several measures of body size, including body weight and height, and body surface area (BSA) [1, 3–5]. These associations have led to the formulation of reference tables or normal ranges for heart mass from easy-to-obtain characteristics and body measures [1, 4–6]. Although being associated with heart mass, these factors are commonly neglected in estimating actual heart mass in scientific literature.

Several reports suggest that addition of indirect heart mass measures to these models could improve the accuracy of heart mass estimation. For instance, non-invasive measures of the left ventricle have been found to adequately estimate both left ventricular mass and measured wall thickness in post-mortem cases [7, 8]. Similarly, left ventricular circumferential area determined from PMCT has been associated with heart mass and found to reasonably predict heart mass [9]. The accuracy of heart mass estimation from these non-invasive measures is, however, moderate, and often based on simple regression outcomes rather than multivariable modelling incorporating other important variables such as sex, age, and body dimensions.

In the present study, we examined the relationship between heart mass physically measured during autopsy and heart volume as determined from PMCT, while also incorporating sex, age, and body dimensions into a multivariable model. In doing so, we aimed to assess the utility of predicting heart mass from PMCT-derived heart volumes, which could be used to improve accuracy of non-invasive heart mass estimations in post-mortem.

## Methods

### Post-mortem cases

Heart mass (autopsy) and heart volume (PMCT) were measured in 87 consecutive adult (≥ 18 years) cases undergoing routine coronial post-mortem examination between November 2018 and April 2021 as part of a 6-month prospective study at Auckland City Hospital, Auckland, New Zealand. The Chief Coroner authorized all post-mortem examinations and approved the study. Provided that no individuals could be identified by information provided by the forensic pathologist, ethical review by the University of Otago Human Ethics Committee was deemed not required for this study. The age, sex, cause of death, and body weight and height (measured on admission using calibrated scales and inextensible tapes) were available for each post-mortem case. Body mass index (BMI) (body weight [kg] / body height [m]^2^) and BSA (DuBois & DuBois formula: [weight (kg)^0.425^ × height (cm)^0.725^] × 0.007184) were calculated from case body weights and heights. Cases were excluded if the heart was anatomically compromised (cases of trauma, decomposition, and hearts that had undergone surgery), grossly adherent to surrounding serosa surfaces, or containing mechanical valves as these pathologies are known to change heart mass significantly. No pediatric or suspicious cases were examined for this study.

### Post-mortem computed tomography

Each post-mortem case underwent a non-contrast CT scan (Siemens SOMATOM Scope, Healthineers AG, Germany) of the head and torso prior to examination. Heart volume was determined by manual tracing of the pericardial sac in several axial slices (3 mm) between the first piece of the superior tip of the right atrial appendage (upper bound) and the visualization of the right coronary artery inferior to the right atrium (lower bound). If the right coronary artery was not visible inferiorly, the inferior atrioventricular groove was used as the lower bound. Measurements were made using 3D Slicer software [10], which interpolated between traced segments. This software is open access and does not require specialized equipment (https://www.slicer.org/).

### Heart mass measurements

Heart mass was measured as described previously [11, 12]. Hearts were removed from the pericardial sac by cutting the great vessels, as prescribed by standardized European and American guidelines [13, 14]. The aorta and pulmonary trunk were cut approximately 30 mm above the semilunar valve and the pulmonary veins were cut at the pericardial reflection. Inferior and superior vena cavae were cut at the diaphragm and 20 mm above the point where the crest of the right atrial appendage meets the superior vena cava, respectively [13]. The heart was dissected using the short axis method recommended by the most recent European guideline [13] before being weighed to give total heart mass. Heart mass included the weight of the chambers, coronary arteries, and small segments of the vascular connections. Blood and blood clots in the heart chambers were totally removed and heart chambers were pad dried prior to measurements. Heart mass was measured using a SW15KM digital scale (A & D Company Ltd., Tokyo, Japan) with a capacity of 6000 g and an error margin of 5 g.

### Statistical analysis

Data are presented as median values with minimum-to-maximum or interquartile ranges. Data normality was assessed using Shapiro–Wilk test. Differences in absolute values of continuous variables between male and female cases were assessed by unpaired *t*-tests or Mann–Whitney *U* tests depending on normality of data distribution. Spearman correlations were performed on untransformed data. Data were analyzed by simple and stepwise multiple linear regression analyses as a total case cohort and when separated as male and female cases. For stepwise linear regression, heart mass was used as the response variable and age, sex (dichotomous, *female* as reference), BSA, and heart volume were used as predictor variables. Only variables with *α* < 0.05 were retained in each final model. All analyses were performed using GraphPad Prism (Version 9.2.0, GraphPad Software Inc., USA) or Minitab (Minitab Statistical Software, USA) software. *P* < 0.05 was considered significant for all analyses.

## Results

### Post-mortem case characteristics

Heart masses and volumes were collected and analyzed from 87 total post-mortem cases (63 male, 24 female). Causes of death of the cases included cardiovascular disease/event (acute myocardial infarction, ischemic heart disease, pulmonary hypertension, hypertensive heart disease, coronary artery disease, valvular heart disease, pulmonary thromboembolism) (*N* = 45), subarachnoid or subdural hemorrhage (*N* = 4), alcohol/drug toxicity (*N* = 12), hanging (*N* = 7), drowning (*N* = 1), asphyxiation (*N* = 2), asthma (*N* = 2), gastrointestinal hemorrhage (*N* = 3), sepsis (*N* = 2), acute pyelonephritis (*N* = 1), diabetic ketoacidosis (*N* = 2), or other (*N* = 6). The self-identified ethnicities of the post-mortem cohort, as retrieved from medical records, included New Zealand European (*N* = 46), Māori (*N* = 20), Pasifika (*N* = 12), Indian (*N* = 6), or non-disclosed Asian ethnicity (*N* = 3).

As shown in Table 1, the median age of the post-mortem cases was 56 years with a minimum age of 18 years and a maximum age of 86 years. There was a wide spread in the body heights (172 cm median, 148–192 cm range) and weights (80 kg median, 34–148 kg range) and, consequently, of the derivative anthropometric indices of BMI and BSA. There was no difference in median age (*P* = 0.26) or BMI (*P* = 0.16) between male and female cases. Male post-mortem cases did, however, have significantly greater median BSA (*P* < 0.0001), body weight (*P* = 0.001), body height (*P* < 0.0001), and heart mass (*P* = 0.0008) and heart volume (*P* = 0.008) than female cases.

**Table 1:** Post-mortem case characteristics

Variable	All cases	Males	Females	*P* value
Sample	87	63	24	
Age (years)	56 (18–86)	55 (18–78)	63 (23–86)	0.26
BMI (kg/m^2^)	28.0 (13.0–48.9)	28.4 (18.7–48.9)	26.7 (13.0–40.3)	0.16
BSA (m^2^)	2.0 (1.3–2.6)	2.0 (1.4–2.6)	1.8 (1.3–2.3)	< 0.0001
Body weight (kg)	80 (34–148)	86 (49–148)	72 (34–120)	0.001
Body height (cm)	172 (148–192)	176 (148–192)	163 (148–175)	< 0.0001
Heart mass (g)	435 (215–865)	465 (270–865)	388 (215–580)	0.0008
Heart volume (cm^3^)	736 (282–1495)	770 (324–1495)	574 (282–1026)	0.008

For all variables (including heart mass and heart volume, data are not transformed). Differences in normally distributed data (age, BMI, BSA, body weight, and height) were determined by unpaired *t*-test. Differences in non-normally distributed data (heart mass and heart volume) were determined by Mann–Whitney *U* test

*BMI*, body mass index; *BSA*, body surface area

### Univariable analyses of heart mass and PMCT-derived heart volume and formulation of heart mass estimation equation from simple linear regression

Spearman correlation analyses of the untransformed data of the total cohort showed that heart mass and heart volume were not associated with age, while significant univariable associations were found for each heart measure with female sex (mass *r*_*s*_ [Spearman rho] =  −0.356; volume *r*_*s*_ =  −0.285), BMI (mass *r*_*s*_ = 0.643; volume *r*_*s*_ = 0.573), BSA (mass *r*_*s*_ = 0.725; volume *r*_*s*_ = 0.626), and body weight (mass *r*_*s*_ = 0.714; volume *r*_*s*_ = 0.631) (Table 2). Table 2 also shows that BMI, BSA, and body weight were positively associated with heart mass and heart volume in males and females.

**Table 2:** Spearman correlation analyses of heart mass predictors

Variable	All cases		Male cases		Female cases	
	***r***_***s***_	***P* value**	***r***_***s***_	***P* value**	***r***_***s***_	***P* value**
***Heart mass***						
Age	−0.015	0.85	0.040	0.76	0.140	0.52
Sex	−0.356	< 0.0001	-	-	-	-
BMI	0.643	< 0.0001	0.631	< 0.0001	0.645	0.001
BSA	0.725	< 0.0001	0.720	< 0.0001	0.570	0.004
Body weight	0.714	< 0.0001	0.711	< 0.0001	0.618	0.001
***Heart volume***						
Age	−0.030	0.99	0.082	0.53	−0.046	0.83
Sex	−0.285	0.004	-	-	-	-
BMI	0.573	< 0.0001	0.480	< 0.0001	0.682	< 0.0001
BSA	0.626	< 0.0001	0.558	< 0.0001	0.549	0.005
Body weight	0.631	< 0.0001	0.551	< 0.0001	0.623	0.001

Spearman rho coefficient represented by *r*_*s*_. Heart mass and heart volume data are not transformed. All cases *N* = 87, male cases *N* = 63, female cases *N* = 24

Simple linear regression analyses were performed to determine the univariable associations of heart mass with variables to be used in multivariable modelling (Table 3). BSA was the only anthropometric variable included in the analysis because it has been reported to best predict heart mass in the general population [1]. Additionally, inclusion of only BSA limited variable collinearity (with body weight, body height, or BMI) in the later multivariable analyses. Simple linear regression of the total case cohort found that heart mass is significantly associated with female sex (negatively), BSA, and heart volume, with heart volume univariably explaining ~ 72% of total heart mass variability (Table 3). Similar associations of heart mass with BSA and heart volume (males *R*^2^ = 0.70; females *R*^2^ = 0.64) were found in male and female cases when analyzed separately. The following equations derived from these simple linear regression analyses were used to estimate heart mass (estimates herein referred to as *Simple*):

**Table 3:** Simple linear regression analyses of heart mass predictors

Predictor variable	All cases			Male cases			Female cases		
	***β***	***P* value**	***R***^**2**^	***β***	***P* value**	***R***^**2**^	***β***	***P* value**	***R***^**2**^
Age	0.938	0.738	0.00	1.319	0.628	0.00	1.045	0.476	0.02
Sex	−102.9	0.0009	0.12	-	-	-	-	-	-
BSA	310.8	< 0.0001	0.46	331.0	< 0.0001	0.42	214.8	0.0048	0.31
Heart volume	0.456	< 0.0001	0.72	0.66	< 0.0001	0.70	0.385	< 0.0001	0.64

Data were analyzed by simple linear regression of relationship with heart mass. *BSA*, body surface area. Sex = female sex as reference. All cases *N* = 87, male cases *N* = 63, female cases *N* = 24

$$\begin{aligned}&Total\;case\;cohort\;heart\;mass\;(g)\\&=\:127.3\:+\:0.4556\;\ast\;heart\;volume\end{aligned}$$

$$\begin{aligned}&Male\;case\;cohort\;heart\;mass\;(g)\\&=\:138.4\:+\:0.4525\;\ast\;heart\;volume\end{aligned}$$

$$\begin{aligned}&Female\;case\;cohort\;heart\;mass\;(g)\\&=\:148.1\:+\:0.3845\;\ast\;heart\;volume\end{aligned}$$

### Multivariable analyses of heart mass predictors and formulation of regression equations for estimating heart mass

Multivariable linear regression analysis was then used to identify independency of predictors and to generate additional and more adequate equations for estimating heart mass. Predictors of heart mass were determined from two models. *Model 1* incorporated post-mortem case age, sex, and BSA, while *model 2* incorporated PMCT-derived heart volume alongside case age, sex, and BSA. As shown in Table 4, *model 1* found that heart mass is independently associated with age and BSA in the total case cohort, with the multivariable model predicting 54% of the variation in heart mass. Age and BSA were also independently associated with heart mass in male and female cases when analyzed separately, with each model predicting 46% and 53% of heart mass variation, respectively (Table 4).

**Table 4:** Multivariable regression analyses of heart mass predictors

Predictor variable	All cases			Male cases			Female cases		
	***β***	***P* value**	***R***^**2**^** adj**	***β***	***P* value**	***R***^**2**^** adj**	***β***	***P* value**	***R***^**2**^** adj**
***Model 1***									
Age	2.641	0.0002	**0.54**	2.256	0.027	**0.46**	2.941	0.002	**0.53**
Sex				-	-		-	-	
BSA	359.8	< 0.0001		356.1	< 0.0001		332.4	< 0.0001	
***Model 2***									
Age	1.344	0.008	**0.78**			**0.74**	2.104	0.004	**0.74**
Sex				-	-		-	-	
BSA	147.7	< 0.0001		113.5	0.008		168.8	0.012	
Heart volume	0.345	< 0.0001		0.380	< 0.0001		0.279	0.002	

Data were analyzed by stepwise linear regression (*α* < 0.05 to enter model and > 0.05 to leave model). *Model 1* incorporated age and body surface area (BSA) as predictor variables for heart mass. *Model 2* incorporated age BSA, and heart volume measured from post-mortem computed tomography as predictors. Sex = female sex as reference. “Adj” = adjusted. All cases *N* = 87, male cases *N* = 63, female cases *N* = 24

Regression analysis using *model 2* (inclusion of PMCT-derived heart volume) found that heart volume is independently predictive of heart mass alongside age and BSA (Table 4). Interestingly, female sex was not independently associated with heart mass in this model. Addition of heart volume to the model notably increased the adjusted *R*^2^ relative to that found with *model 1* and with the *Simple* univariable regression (*R*^2^ = 0.72, Table 3), suggesting that *model 2* can predict up to 78% of variation in post-mortem heart mass. In male cases, inclusion of heart volume in the model revealed that heart mass is independently associated with BSA and heart volume, but not with age. In female cases, age, BSA, and heart volume were independently associated with heart mass. In both male and female cases, *model 2* was notably improved in heart mass predictive capability compared to *model 1* and the *Simple* univariable model (male cases *R*^2^ adjusted = 0.74; female cases *R*^2^ adjusted = 0.74).

These regression analyses indicate that heart volume measured from PMCT is independently associated with heart mass at univariable and multivariable levels. Moreover, addition of age and BSA with heart volume to prediction models improves heart mass estimation accuracy in the total cohort and after sex separation. The regression equations generated from *models 1* and *2* were then used to estimate heart mass in the post-mortem case cohort.

The following equations derived from *model 1* were used to estimate heart mass:

$$Total\;case\;cohort\;heart\;mass\;(g)\:=\:\:-\:398.0\:+\:2.641\;\ast\;age\:+\:359.8\;\ast\;BSA$$

$$Male\;case\;cohort\;heart\;mass\;(g)\:=\:\:-\:366.0\:+\:2.256\;\ast\;age\:+\:356.1\;\ast\;BSA$$

$$Female\;case\;cohort\;heart\;mass\;(g)\:=\:\:-\:376.0\:+\:2.941\;\ast\;age\:+\:332.4\;\ast\;BSA$$

The following equations derived from *model 2* were used to estimate heart mass:

$$Total\;case\;cohort\;heart\;mass\;(g)\:=\:\:-\:159.1\:+\:1.344\;\ast\;age\:+\:147.7\;\ast\;BSA\:+\:0.3469\;\ast\;heart\;volume$$

$$Male\;case\;cohort\;heart\;mass\;(g)\:=\:\:-\:37.3\:+\:113.5\;\ast\;BSA\:+\:0.3804\;\ast\;heart\;volume$$

$$Female\;case\;cohort\;heart\;mass\;(g)\:=\:\:-\:141.9\:+\:1.750\;\ast\;age\:+\:139.7\;\ast\;BSA\:+\:0.2896\;\ast\;heart\;volume$$

### Evaluating performance of estimated heart mass equations

**Fig. 1:**
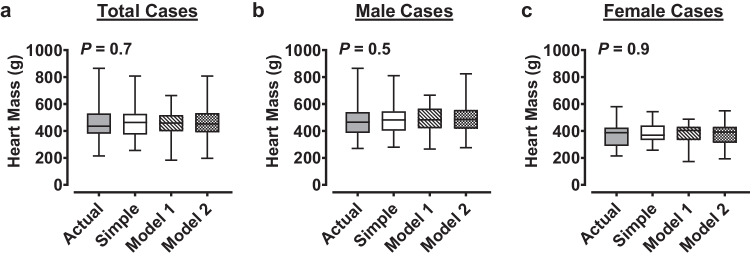
Comparison of heart masses estimated from the univariable and multivariable regression equations. Heart masses were estimated from regression equations calculated from simple linear regression (*Simple*) or stepwise linear regression (*model 1* and *model 2*, see main text for equations). *Simple* model estimated heart mass univariably from heart volume measured from post-mortem computed tomography. *Model 1* estimated heart mass from age and BSA for each analysis group (total cases, male cases, female cases). *Model 2* estimated heart mass from age, BSA, and heart volume measured from post-mortem computed tomography. Median (with maximum and minimum) values for each estimation equation and comparison to median actual heart mass for total case cohort and male and female cases are shown in **a**, **b**, and **c**, respectively. Total cases *N* = 87, male cases *N* = 63, female cases *N* = 24

**Fig. 2:**
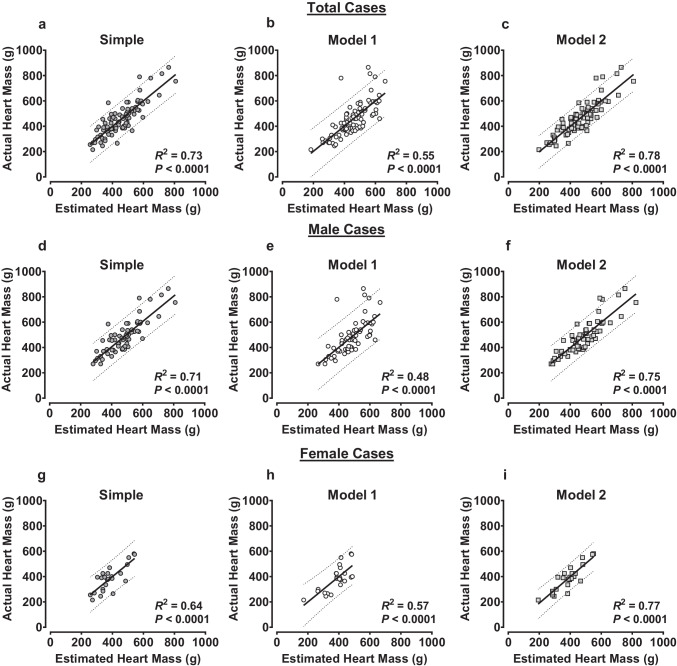
Performance of heart mass estimates generated from univariable and multivariable regression equations against actual heart mass. Heart masses were estimated from regression equations calculated from simple linear regression (*Simple*) or stepwise linear regression (*model 1* and *model 2*, see main text for equations). *Simple* model estimated heart mass univariably from heart volume measured from post-mortem computed tomography. *Model 1* estimated heart mass from age and BSA for each analysis group (total cases, male cases, female cases). *Model 2* estimated heart mass from age, BSA, and heart volume measured from post-mortem computed tomography. Simple linear regressions of actual heart mass vs. heart mass estimated from the univariable *Simple* model, multivariable *model 1*, or multivariable *model 2* of the total case cohort are shown in **a**–**c**. Equivalent regressions from male cases or female cases are only shown in **d**–**f** or **g**–**i**, respectively. Data are presented with regression line and 95% prediction bands. Total cases *N* = 87, male cases *N* = 63, female cases *N* = 24

We then determined the capacity of each of the estimates generated from univariable and multivariable analyses to predict actual heart mass. The median heart mass values calculated from the *Simple* regression model (463 ± 154 g [interquartile range]) and multivariable *model 1* (458 ± 121 g) and *model 2* (452 ± 143 g) were not different to the median actual heart mass (435 ± 150 g) in the total cohort (Friedman test *P* = 0.7) (Fig. 1a). This result was consistent when analyzed as male (*P* = 0.5) or female (*P* = 0.9) cases only (Fig. 1b + c). When analyzed by simple linear regression, heart mass estimated from the *Simple* regression equation predicted 73%, 71%, and 64% of actual heart mass variation in the total cohort (Fig. 2a), male cases (Fig. 2d), and female cases (Fig. 2g), respectively. As expected, this recapitulated the *R*^2^ values found by simple linear regression of actual heart mass vs. actual heart volume (Table 3). By comparison, heart mass estimates generated from the multivariable *model 1* (incorporating age and BSA) predicted only 55%, 48%, and 57% of actual heart mass variation in the total cases (Fig. 2b), male cases (Fig. 2e), and female cases (Fig. 2h), respectively.

Inclusion of PMCT-derived heart volume in the multivariable model (*model 2*) notably improved the predictive capacity of the model. In the total case cohort, heart mass estimated from *model 2* predicted 78% of heart mass variability (Fig. 2c), while in male (Fig. 2f) and female cases (Fig. 2i), up to 75% and 77% could be predicted, respectively. These findings suggest that inclusion of heart volume in heart mass estimation models improves the accuracy of actual heart mass prediction in male and female post-mortem cases.

**Fig. 3:**
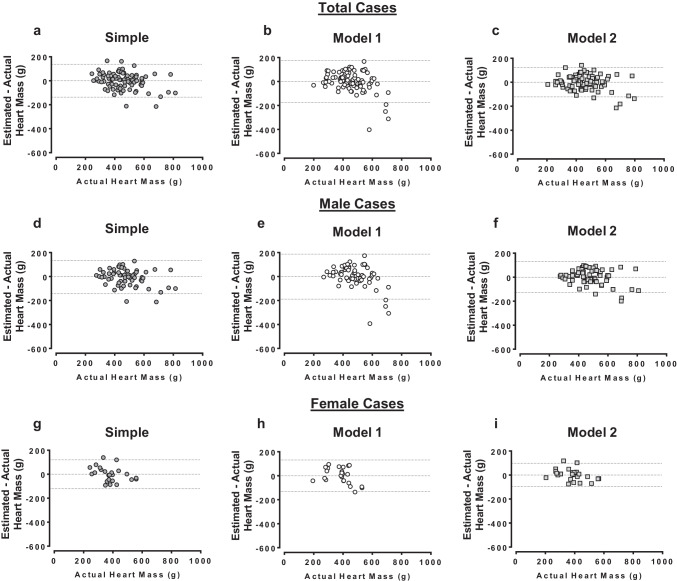
Bland–Altman plots of actual heart mass vs. heart mass estimated from univariable and multivariable regression equations. Heart masses were estimated from regression equations calculated from simple linear regression (*Simple*) or stepwise linear regression (*model 1* and *model 2*, see main text for equations). *Simple* model estimated heart mass univariably from heart volume measured from post-mortem computed tomography. *Model 1* estimated heart mass from age and BSA for each analysis group (total cases, male cases, female cases). *Model 2* estimated heart mass from age, BSA, and heart volume measured from post-mortem computed tomography. All plots show performance of estimated heart mass values relative to difference of the estimated and actual heart mass values. Performance of heart mass estimates from each estimate equation in the total case cohort is shown in **a**–**c**. Similarly, performance of heart mass estimates from each equation in male and female cases only is shown in **d**–**f** or **g**–**i**, respectively. Total cases *N* = 87, male cases *N* = 63, female cases *N* = 24

Finally, the performances of the heart mass estimates from the *Simple* model and multivariable *model 1* and *model 2* were assessed qualitatively by Bland–Altman plots (Fig. 3). In the total cases (Fig. 3a–c), male cases (Fig. 3d–f), and female cases (Fig. 3g–i), the estimates calculated from all models distributed relatively evenly around zero, especially at or below the median heart mass. In each case, larger physical heart masses tended to be underestimated by each set of estimates; however, this effect was most apparent in estimates generated from *model 1*. These data further suggest that addition of PMCT-derived heart volume to models containing other post-mortem case characteristics improves the accuracy of physical heart mass estimates, especially in individuals with greater heart mass where this value is of utmost importance for determining the cause of death.

## Discussion

This study aimed to examine the relationship between heart mass and heart volume measured by PMCT in post-mortem cases. In doing so, we sought to determine whether heart volumes measured non-invasively at post-mortem are a useful surrogate for physically measuring heart mass by invasive autopsy. We found that physical heart mass is independently predicted by heart volume and that heart mass can be estimated reliably from heart volume using sex-specific equations generated from regression modelling. These findings suggest that heart mass can be estimated from PMCT-derived heart volume with greater accuracy when sex, age, and body dimensions are incorporated together with more advanced modelling.

### Inclusion of PMCT-derived heart volume with conventional heart mass predictors improves heart mass estimation accuracy

Post-mortem heart mass has been estimated with varying levels of univariable predictive strength from left ventricular area measured by cMRI (*R*^2^ = 0.78) or left ventricular circumferential area measured by PMCT (*R*^2^ = 0.61) [9, 15]. Additionally, heart mass has been moderately associated with heart mass estimated from a combination of PMCT-derived heart volume and PMCT attenuation (*R*^2^ = 0.53) [7]. In several of the aforementioned studies, heart mass estimates calculated from univariable regression equations of left ventricular dimension were not different to actual heart mass [9, 15]. Importantly, Bland–Altman analyses, when performed [9], revealed these regression-based estimates perform poorly at the upper and lower bounds of heart masses, indicating that further development of heart mass prediction models is still required.

The current study has addressed this by showing that heart mass can be predicted with improved accuracy by sex-specific multivariable regression equations that include PMCT-derived heart volume alongside age and BSA (*model 2*). We found that the estimated heart mass values could account for 78%, 75%, and 77% of actual heart mass variation in the total cohort, in male cases, and in female cases, respectively (Fig. 2). This was a noticeable improvement in predictive capacity compared to the heart mass estimates generated from models accounting only for age and BSA (*model 1*) and from models generated using heart volume alone (*Simple*). Moreover, we found the heart mass calculated from *model 2* can estimate with greater accuracy than *model 1* estimates across the spectrum of actual heart mass values, as indicated by the Bland–Altman analyses in Fig. 3. Together, the improved accuracy of heart mass estimation provided by the multivariable model that incorporates age, BSA, and heart volume (*model 2*) should facilitate more sensitive diagnosis of abnormal heart mass levels when compared to normal reference ranges [1, 4, 5].

With regard to a previous study of heart mass estimation from PMCT (Ogawa et al. [7]), the improved estimation accuracy found in the current study likely arises from the stepwise multivariable integration of PMCT-derived heart volume measures with age, sex, and BSA that can be afforded by a larger sample size. As age, sex, and body size are already used to estimate normal heart mass, adding these factors alongside heart volume in the model will generate a more direct snapshot of the heart that is absent in estimation models based on case characteristics and body size measurements alone. This notion is supported by univariable regression analysis in the current study, which found that heart volume alone can explain up to 64–72% of heart mass variability, while BSA alone can explain only 31–46% (Table 3).

### Implementation of new estimates against normal reference standards

Several reference guides for estimating normal heart mass from post-mortem case characteristics have been established [1, 4, 5]. This includes that of Wingren and Ottosson, which provides normal heart mass reference ranges generated from > 27,000 non-natural post-mortem cases (i.e., cause of death not directly due to disease) [4]. As a proof of concept, we also compared the median heart mass values generated from the multivariable model including PMCT from the current study (*model 2*) with heart mass values generated from the Wingren and Ottosson normal reference guide (data not shown). In each analysis group (total, male, and female cases), both the actual and estimated heart masses from this study were significantly greater than the heart mass values estimated by the reference guide calculations. Additionally, when assessed as a predictor of the actual heart mass values of the current study, the heart mass estimates generated from the reference guide performed modestly (*R*^2^ = 0.55 in total cohort). This post hoc analysis highlights two important aspects. Firstly, the current study includes a high proportion of likely pathologically hypertrophied hearts, as is evident in the high percentage of cardiac-related causes of death (52%). Secondly, this analysis indicates that the heart mass estimation equation proposed here can be used to identify abnormal heart masses when used in conjunction with normal heart mass reference guides.

### Limitations

This study was limited in several aspects. Firstly, as described above, our data indicate that the post-mortem cases used in the study do not represent those with “normal” heart mass. Therefore, caution is required when generalizing the findings to a wider non-forensic population. It will be necessary in future work to examine the relationship between PMCT-derived heart volume (together with age and BSA) with heart mass in otherwise “healthy” post-mortem cases and to test our calculations on pre-existing CT scans gathered through the lifetime of patients. Secondly, the key measures of heart mass and heart volume were performed by only a single forensic pathologist or researcher, respectively, potentially introducing some bias in the data collection processes. Importantly, heart volume was measured by a standard method based on easy to identify landmarks, while the heart mass was measured using a thorough and validated method that removes the confounding influence of water residual from rinsing [11, 12]. Finally, previous study of heart mass predictors in a multi-ethnic Aotearoa New Zealand post-mortem cohort found that heart mass can be independently predicted by ethnicity (as a dichotomous variable) when modelled alongside age, male sex, and body size indices [3]. Case ethnicity, as self-identified in medical records, was not considered as a variable in the current study due to insufficient sample size. However, given that left ventricular mass indexation to body size performs differently in Māori and Pacific New Zealanders [16], further evaluation of whether ethnicity changes heart mass estimation from PMCT-derived heart volume is required in the future.

## Conclusions

Using physical mass and non-invasive imaging measures from post-mortem cases, we show that heart volume, as derived from PMCT, can independently predict heart mass in male and female cases when incorporated into estimation models alongside age, sex, and body size measurement. This suggests that, while further research is required to validate these equations in other cohorts, heart volume measured from PMCT is a useful surrogate for physical heart mass, which can then be used to calculate estimated heart mass for comparison to normal heart mass reference guides.

## Key points

1. Estimating heart mass using heart volume measured from PMCT alone has limited accuracy.
2. Heart mass can be estimated from case characteristics, such as age, sex, and body dimensions; however, these were not incorporated in heart mass estimation when using heart volume in previous studies.
3. We determined whether heart mass estimation can be improved when age, sex, and body dimensions were incorporated with heart volume.
4. Heart volume predicted 72% of heart mass variation univariably, whereas multivariable regression incorporating age and body surface area explained up to 78%.
5. Heart mass can be more accurately predicted from non-invasively measured heart volume when modelled with other classical heart mass predictors.
